# Longitudinal Visual Evoked Potential Changes Show Distinct Associations with Relapse Activity and Disability in Relapsing–Remitting Multiple Sclerosis: A Multicenter Retrospective Cohort Study

**DOI:** 10.3390/medicina62091750

**Published:** 2026-09-11

**Authors:** Samet Öncel, Meral Seferoğlu, Sami Ömerhoca, Semanur Aksu, Ertuğrul Çınar, Hakan Kılıçaslan, Abdulkadir Tunç, Nilüfer Kale İçen

**Affiliations:** 1 Department of Neurology, Sakarya Training and Research Hospital, 54100 Sakarya, Turkey; sametoncel@yahoo.com (S.Ö.); semanuraksu54@gmail.com (S.A.); 2 Department of Neurology, Bursa Yüksek İhtisas Training and Research Hospital, 16310 Bursa, Turkey; meralbozseferoglu@gmail.com (M.S.); hkilicaslan2306@gmail.com (H.K.); 3 Department of Neurology, Bağcılar Training and Research Hospital, 34200 Istanbul, Turkey; samiyumerhodzha@yahoo.com (S.Ö.); drertugrulcinar@gmail.com (E.Ç.); kalenilufer@yahoo.com (N.K.İ.); 4 Department of Neurology, Faculty of Medicine, Sakarya University, 54100 Sakarya, Turkey; 5 Bloocell Health Technologies Industry and Trade Joint Stock Company, 54580 Sakarya, Turkey

**Keywords:** multiple sclerosis, visual evoked potentials, neurodegeneration, disease progression, prognosis

## Abstract

*Background and Objectives*: Visual evoked potentials (VEPs) are established tools for detecting optic pathway involvement in multiple sclerosis (MS); however, the distinct clinical significance of longitudinal changes in latency and amplitude remains unclear. This exploratory study aimed to evaluate the clinical utility of serial VEP assessments in patients with relapsing–remitting MS (RRMS) by examining the associations between temporal changes in latency and amplitude and concurrent markers of disease activity and disability. *Materials and Methods*: We retrospectively analyzed 83 patients with RRMS from three centers who underwent at least two pattern-reversal VEP assessments ≥ 1 year apart. Patients with recent optic neuritis were excluded. Longitudinal changes in VEP latency and amplitude were evaluated in relation to relapse activity during the same inter-assessment interval, baseline MRI and CSF findings, and disability assessed by the Expanded Disability Status Scale (EDSS). Sensitivity analyses were stratified and adjusted for disease-modifying therapy (DMT) efficacy tier, and progression independent of relapse activity (PIRA) was examined using a formal definition. *Results*: Over a median follow-up of 2 years, 43.4% of patients developed P100 latency prolongation, which was independently associated with a higher annualized relapse rate during the same interval (odds ratio [OR] 2.91, *p* = 0.005; area under the curve (AUC) 0.72 for concurrent on-study relapse activity), an association that persisted after adjustment for DMT efficacy tier, but not with disability worsening. In contrast, 56.6% of patients exhibited amplitude reduction, which was associated with greater disability accumulation at the group level, with higher EDSS scores at follow-up despite similar baseline values (*p* < 0.05); the corresponding inverse correlation between amplitude decline and EDSS change was only nominally significant (ρ = −0.231, *p* = 0.036), did not survive correction for multiple comparisons, and was attenuated after adjustment for baseline EDSS. Formally defined PIRA events were rare (*n* = 3), precluding inference. Baseline magnetic resonance imaging (MRI) and cerebrospinal fluid (CSF) findings differed in the latency group, with fewer MRI lesions and a lower immunoglobulin G (IgG) index, whereas no corresponding differences were observed in the amplitude group. *Conclusions*: In this exploratory cohort, longitudinal prolongation of VEP latency was associated with concurrent relapse activity, whereas amplitude decline showed a weaker, group-level association with disability. These findings are consistent with, but do not demonstrate, distinct inflammatory and neurodegenerative substrates, and warrant prospective validation before serial VEP monitoring can inform individualized management in MS.

## 1. Introduction

Multiple sclerosis (MS) is a chronic inflammatory demyelinating disease of the central nervous system that frequently involves the visual pathway. Optic neuritis (ON) is a common manifestation of MS, occurring in up to 50–70% of patients during the disease course [1,2]. Even in the absence of overt ON, subclinical demyelination of the optic nerve and visual pathways is often present in MS, contributing to subtle visual dysfunction that may be unrecognised [3,4,5]. Because the visual system can be readily evaluated using both structural and functional measures, it has emerged as a useful model for studying MS-related neuroinflammation and neurodegeneration in vivo [4,6]. In particular, damage to the anterior visual pathway provides insight into broader MS pathology, given that retinal axons and optic nerve myelin are frequently targets of the disease from its earliest stages [7].

Visual evoked potentials (VEPs) are well-established non-invasive neurophysiological tools for assessing the integrity of visual pathways [8]. In MS, conventional full-field pattern-reversal VEPs have long been used to detect optic nerve lesions that may be clinically silent, thereby providing evidence of dissemination in space, even when patients have no visual symptoms [9,10]. The P100 component of pattern-reversal VEP, a positive waveform peaking at approximately 100 ms, is of particular clinical relevance. Delayed P100 latency in patients with MS is highly sensitive to optic nerve demyelination or incomplete remyelination and can be observed even in eyes without a history of acute ON [8,11]. Thus, VEPs serve as an objective means to uncover subclinical visual pathway involvement in MS, complementing other diagnostic modalities, such as MRI. The diagnostic relevance of VEP has recently been reaffirmed by the 2024 revision of the McDonald criteria, which for the first time incorporates the optic nerve as a fifth central nervous system topography and endorses VEP as objective evidence of optic nerve involvement for demonstrating dissemination in space [12]. In addition to their diagnostic utility, VEPs are increasingly recognized for their potential use in monitoring diseases and as outcome measures in clinical trials aimed at neuroprotection or repair [7,9]. Cross-sectional studies have demonstrated that prolongation of VEP latency correlates with greater MRI lesion load and even with brain atrophy, suggesting that slower visual conduction may reflect a broader impact of MS on central nervous system integrity [4,13]. VEP abnormalities have also been associated with clinical disability; for example, patients with prolonged P100 latencies or reduced amplitudes tend to have higher Expanded Disability Status Scale (EDSS) scores and worse functional outcomes [3,14]. Longitudinal assessment of VEPs could offer a means to track subclinical disease progression in MS; however, to date, only a few studies have addressed this in detail [3,6,7]. Earlier investigations suggested that serial evoked potential testing might predict future outcomes. For example, Fuhr et al. [15] reported that VEP and motor EP changes over time paralleled the clinical evolution of MS, and Kallmann et al. [16] reported that patients with abnormal baseline EPs developed greater disability during follow-up. Despite these examples, no consensus has been reached on how to incorporate serial VEP monitoring into routine practice, largely because robust longitudinal data linking VEP changes to definitive clinical or radiological outcomes are limited. In particular, the question remains whether progressive latency prolongation or amplitude loss on VEP truly anticipates an upcoming disability or MRI changes, or whether these measures simply mirror damage that has already occurred.

This exploratory study aimed to assess the clinical utility of longitudinal VEP measurements in relapsing–remitting MS (RRMS), focusing on whether latency and amplitude changes show distinct associations with concurrent inflammatory activity and disability.

## 2. Materials and Methods

### 2.1. Study Design and Participants

**Figure 1:**
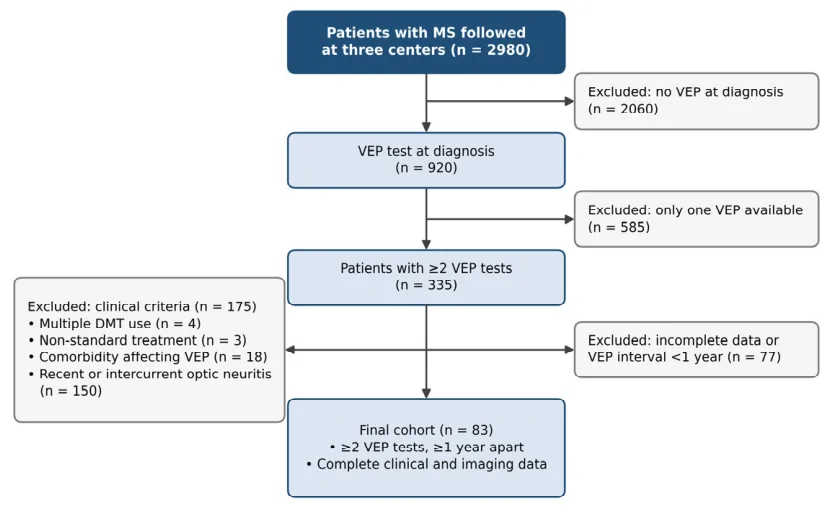
Patient selection flowchart. Flowchart illustrating the selection process of patients included in the study. Of 2980 patients with MS followed across three centers, 920 underwent VEP testing at diagnosis. Following exclusion based on the availability of serial VEPs and the clinical eligibility criteria, 83 patients with at least two VEP assessments spaced ≥ 1 year apart and complete clinical/imaging data were included in the final analysis.

This multicenter retrospective cohort study was conducted using data from three MS centers in Turkey: Sakarya Training and Research Hospital (Sakarya), Bursa Yüksek İhtisas Training and Research Hospital (Bursa), and Bağcılar Training and Research Hospital (Istanbul). All patients were diagnosed with RRMS according to the 2017 revised McDonald criteria [17]. In accordance with national regulations for multicenter retrospective studies, institutional permission was first obtained from the administration of each participating hospital, after which a single ethics approval covering all three centers was granted by the Sakarya University Faculty of Medicine Ethics Committee (approval number: 150, 19 November 2024). The study was conducted in accordance with the Declaration of Helsinki. Owing to the retrospective design of the study, the requirement for written informed consent was waived by the Ethics Committee of Sakarya University Faculty of Medicine. From an initial pool of 2980 screened records, 83 patients met the inclusion criteria: age 18–65 years, at least two full-field pattern-reversal VEP assessments performed ≥1 year apart, and complete clinical and radiological data. Exclusion criteria were as follows: comorbid conditions affecting visual or neurological function (e.g., diabetes mellitus, Graves’ disease, uveitis), baseline EDSS score > 5.5, incomplete clinical or imaging data, use of nonstandard immunosuppressive therapies or multiple disease-modifying treatments within 6 months before or between VEP assessments, MS relapse or suspected optic neuritis within 3 months prior to VEP testing, and any episode of optic neuritis occurring between the two VEP assessments. The patient selection process is illustrated in Figure 1.

### 2.2. Clinical and Radiological Data Collection

Demographic variables included age and sex. Clinical variables comprised disease duration, EDSS score, and annualized relapse rate (ARR). ARR was calculated as the number of clinically confirmed relapses occurring between the first and second VEP assessments divided by the length of that interval in years; thus, ARR and longitudinal VEP changes refer to the same observation window, and the associations reported are concurrent rather than predictive. EDSS was recorded at the first VEP, at the second VEP, and at the last available clinical visit. Treatment type and duration during the interval between VEP assessments were also recorded. Disease-modifying therapies (DMTs) used during this interval were grouped, in line with published efficacy classifications and the ECTRIMS/EAN guideline framework [18,19], as platform or moderate-efficacy (interferon-β, glatiramer acetate, teriflunomide, dimethyl fumarate) or high-efficacy (fingolimod, natalizumab, cladribine, ocrelizumab) agents. Baseline MRI variables included the presence of lesions in the brainstem, spinal cord, and upper cervical cord (C1–C4), total T2 lesion burden, and the presence of gadolinium-enhancing lesions. Brain and spinal cord MRI at all three centers were acquired on 1.5 Tesla MRI systems (GE Healthcare, Chicago, IL, USA) using a standardized MS protocol comprising axial and sagittal T2-weighted/FLAIR sequences and pre- and post-gadolinium T1-weighted sequences; the same field strength and acquisition protocol were used across sites, and images were rated by the treating neurologists at each center. Baseline MRI was the diagnostic scan performed within a maximum of 6 months of the first VEP, and baseline MRI data were available for all 83 included patients, as complete imaging data were an inclusion criterion. Cerebrospinal fluid (CSF) analysis, performed at diagnosis within the same time window, included oligoclonal band (OCB) status and IgG index; CSF data were available in 72 patients.

### 2.3. Visual Evoked Potentials (VEPs)

VEP assessments were performed routinely, independent of clinical suspicion of relapse or optic neuritis, using a Keypoint Classic electromyography/evoked potential system (Alpine Biomed ApS, Skovlunde, Denmark). All three centers used identical devices and standardized stimulus displays in accordance with a shared protocol based on the International Society for Clinical Electrophysiology of Vision (ISCEV) guidelines, ensuring consistency in stimulus presentation and recording across sites [8].

Pattern-reversal stimulation was delivered using a high-contrast checkerboard pattern (8 × 8 checks), with each check subtending approximately 1° of visual angle at a viewing distance of 100 cm. Recordings were obtained under controlled conditions, including standardized ambient lighting, room temperature (22–24 °C), and stable fixation. Electrodes were placed according to the international 10–20 EEG system, with the active electrode at Oz, reference at Fz, and ground at Cz. Monocular full-field stimulation was applied, and P100 latency and N75-P100 peak-to-peak amplitudes were measured bilaterally.

Given the retrospective design and absence of a locally derived normative dataset, abnormality thresholds were defined based on ISCEV-compliant reference values. A P100 latency > 118 ms and amplitude < 5 µV were considered abnormal [8,20]; these cut-offs defined cross-sectional (single-timepoint) abnormality. Separately, longitudinal change between the two assessments was evaluated: latency prolongation was defined as an increase of ≥2 ms from baseline in at least one eye (a longitudinal-change threshold, distinct from the cross-sectional cut-off above), whereas amplitude reduction was defined as a decrease of >0.3 µV from baseline or a value below 5 µV in at least one eye. Latency and amplitude abnormalities were assessed at the individual-eye level, and a patient was classified as showing latency prolongation or amplitude reduction if either eye met the respective threshold; the two classifications were defined independently and were not mutually exclusive. Associations between longitudinal changes in VEP parameters (latency and amplitude) and disability outcomes were evaluated using correlation analyses.

### 2.4. Statistical Analysis

Statistical analyses were performed using IBM SPSS Statistics for MacOS (version 30.0; IBM Corp., Armonk, NY, USA). Categorical variables are reported as frequencies and percentages, and continuous variables as medians (range). The Kolmogorov–Smirnov test was used to assess normality. Mann–Whitney U tests were used to compare continuous variables, and Pearson’s chi-square or Fisher’s exact tests were used to compare categorical variables. Spearman’s rank correlation was used to evaluate the relationship between the VEP changes and EDSS worsening. Logistic regression analysis was performed to identify factors independently associated with latency prolongation. The discriminative ability of longitudinal latency change for concurrent on-study relapse activity (ARR > 0 during the inter-assessment interval) was assessed by receiver-operating-characteristic (ROC) analysis, reporting the area under the curve (AUC) with bootstrap 95% confidence intervals. To account for the non-independence of the two eyes, a generalized estimating equation (GEE) logistic model with an exchangeable working correlation was fitted at the eye level. Between-group differences in ordinal outcomes were additionally quantified using Hodges–Lehmann median differences with 95% confidence intervals. Confirmed disability worsening was defined a priori as an EDSS increase of ≥1.5 points from a baseline of 0, ≥1.0 point from a baseline of 1.0–5.5, or ≥0.5 points from a baseline > 5.5 at the second VEP, sustained at the last available visit; progression independent of relapse activity (PIRA) was defined as confirmed disability worsening in the absence of any relapse during the inter-assessment interval, and relapse-associated worsening (RAW) as confirmed worsening with at least one relapse, following established definitions [21,22]. To address potential confounding by treatment, sensitivity analyses were performed in which (i) patients receiving moderate-efficacy versus high-efficacy DMT were compared, (ii) the principal associations were re-examined within each DMT stratum, and (iii) DMT efficacy tier was entered as a covariate in the logistic, GEE, and linear regression models. Statistical significance was defined as a two-tailed *p*-value of <0.05. Because of the exploratory nature of the study, results of secondary and sensitivity analyses are reported without adjustment unless stated otherwise and should be interpreted as hypothesis-generating.

## 3. Results

### 3.1. Demographic and Baseline Characteristics

A total of 83 patients with RRMS were included, of whom 79.5% were female, with a median age of 36 years (range, 18–60). The median disease duration was 6.0 years, and the median baseline EDSS score was 1.5 (range, 0–5.5). The median interval between the initial and follow-up VEP assessments was 2.0 years, during which the median ARR was 0.3. At baseline, median P100 latency was 116 ms in the right eye and 114 ms in the left eye, with corresponding amplitudes of 9.0 µV and 9.5 µV. Baseline MRI revealed brainstem lesions in 32.5% of patients, spinal cord lesions in 57.8%, and gadolinium-enhancing lesions in 38.6%. During the inter-assessment interval, 54 patients (65.1%) received platform or moderate-efficacy therapies—most commonly dimethyl fumarate (22.9%) and teriflunomide (19.3%), 24 patients (28.9%) received high-efficacy therapies (fingolimod, natalizumab, cladribine, or ocrelizumab), and 5 (6.0%) were untreated (Table 1, Supplementary Table S3).

### 3.2. Longitudinal Changes in VEP Latency and Amplitude and Their Distinct Clinical Associations

During follow-up, 36 patients (43.4%) developed P100 latency prolongation in at least one eye. This group exhibited a markedly higher ARR during the same interval compared with those without latency prolongation (*p* < 0.001). Baseline MRI findings revealed a lower lesion burden in the latency prolongation group, including fewer brainstem lesions, spinal cord lesions, and upper cervical cord involvement (*p* = 0.047, *p* = 0.005, and *p* = 0.038, respectively). Gadolinium-enhancing lesions were also less frequent in this group (*p* = 0.004). In parallel, the median CSF IgG index was lower among patients with latency prolongation (*p* = 0.035). No significant differences were observed between groups in terms of disease duration, baseline EDSS score, or EDSS worsening (Table 2).

**Figure 2:**
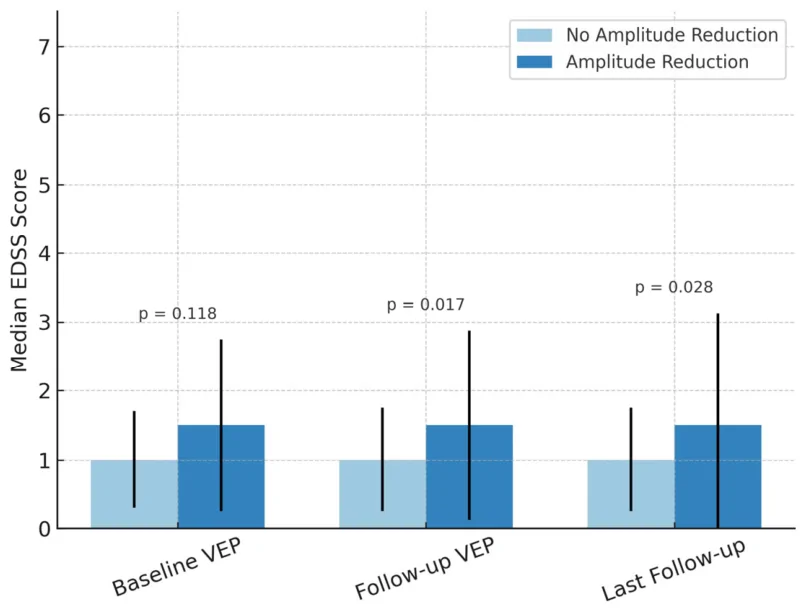
Association between longitudinal amplitude reduction and disability. Bars show median EDSS scores at the baseline VEP, the follow-up VEP, and the last clinical visit in patients with (*n* = 47) and without (*n* = 36) P100 amplitude reduction; error bars indicate the standard deviations. Baseline EDSS did not differ between groups (*p* = 0.118), whereas EDSS was higher in the amplitude-reduction group at the follow-up VEP (*p* = 0.017) and at the last visit (*p* = 0.028; Mann–Whitney U test). The between-group difference was small (0.5 EDSS points), was only nominally significant, and should be interpreted as an exploratory, group-level association.

A total of 47 patients (56.6%) exhibited a significant reduction in P100 amplitude during follow-up. Compared with patients without amplitude reduction, this group had shorter baseline right-eye latency (median 112 ms vs. 119.5 ms, *p* = 0.028), while disease duration and ARR were similar between groups. Despite comparable baseline EDSS scores, patients with amplitude reduction had higher EDSS scores both at the second VEP assessment (*p* = 0.017) and at final follow-up (*p* = 0.028), although the magnitude of the difference was small (0.5 EDSS points). No significant differences were observed between groups in baseline MRI or CSF parameters (Table 3, Figure 2).

### 3.3. Correlation and Multivariate Analyses

**Figure 3:**
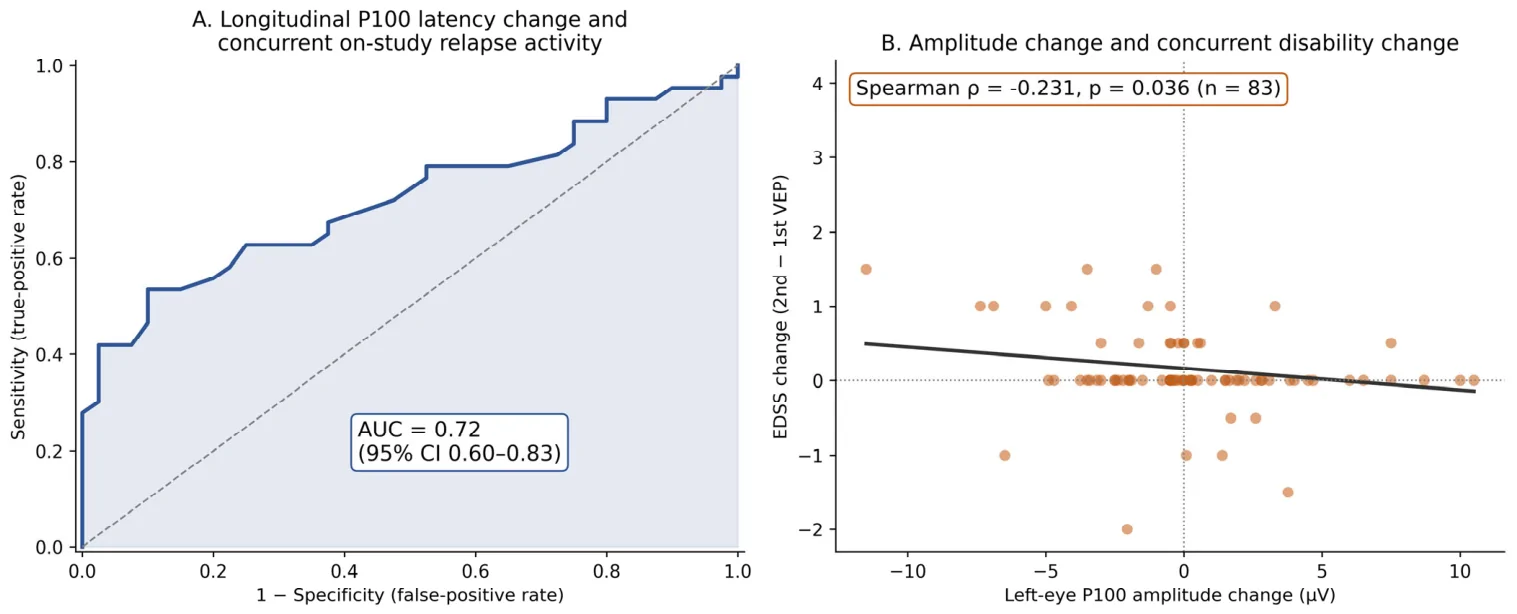
Quantitative performance of longitudinal VEP metrics. (**A**) Receiver-operating-characteristic curve showing that the longitudinal change in mean P100 latency discriminated patients with concurrent on-study relapse activity (annualized relapse rate > 0 during the inter-assessment interval) with an area under the curve of 0.72 (95% CI 0.60–0.83). (**B**) Scatter plot of left-eye P100 amplitude change against the change in EDSS between the first and second VEP assessments, with the fitted regression line; greater amplitude decline was nominally associated with greater concurrent EDSS worsening (Spearman ρ = −0.231, *p* = 0.036, *n* = 83; not significant after Bonferroni correction).

Changes in P100 latency were not significantly correlated with EDSS worsening (all *p* > 0.15). Among eight Spearman correlation tests performed across VEP parameters and time intervals, a nominally significant inverse correlation was observed between left-eye P100 amplitude change and EDSS change between the first and second VEP assessments (*p* = 0.036); however, this association did not survive Bonferroni correction for multiple comparisons, and should therefore be interpreted as an exploratory finding. No significant correlations were identified for other VEP parameters or time intervals (Table 4). Logistic regression analysis including age, disease duration, baseline EDSS score, and ARR identified ARR as the only variable independently associated with latency prolongation (*p* = 0.005). No variables were independently associated with amplitude reduction. In a GEE logistic model accounting for inter-eye correlation, latency prolongation remained associated with the ARR (*p* < 0.001). Longitudinal latency change discriminated patients with concurrent on-study relapse activity with an AUC of 0.72 (95% CI 0.60–0.83), whereas baseline latency did not (AUC 0.52) (Figure 3). Latency prolongation and amplitude reduction overlapped only partially; 23 patients showed both, 13 latency prolongation only, 24 amplitude reduction only, and 23 neither, indicating that the two classifications were largely independent of each other. The higher follow-up EDSS accompanying amplitude reduction corresponded to a Hodges–Lehmann median difference of 0.5 points (95% CI 0.0–0.5), and the higher relapse activity accompanying latency prolongation to a median ARR difference of 0.5 (95% CI 0.2–1.0). In a linear regression model of EDSS at the second VEP adjusted for baseline EDSS, ARR, and DMT efficacy tier, amplitude reduction was associated with a non-significant 0.29-point higher EDSS (95% CI −0.02 to 0.60, *p* = 0.069), indicating that the unadjusted group difference was attenuated after accounting for baseline disability.

### 3.4. Formally Defined Disability Worsening and PIRA

Using the pre-specified definition, confirmed disability worsening between the first and second VEP that was sustained at the last visit occurred in 8 patients (9.6%): 3 (3.6%) fulfilled the criteria for PIRA and 5 (6.0%) for RAW. All three PIRA events and 7 of the 8 confirmed worsening events occurred in patients with amplitude reduction (PIRA: 3/47 vs. 0/36, Fisher’s exact *p* = 0.25; confirmed worsening: 7/47 [14.9%] vs. 1/36 [2.8%], *p* = 0.13), whereas none of the PIRA events occurred in patients with latency prolongation (0/36 vs. 3/47, *p* = 0.25). Among the 40 relapse-free patients, EDSS at the second VEP did not differ significantly between those with and without amplitude reduction (*p* = 0.068), and the amplitude–EDSS correlation was not significant in this subgroup (*p* = 0.31). Given the very small number of events, these analyses are descriptive only and do not permit inference regarding amplitude reduction as a marker of PIRA.

### 3.5. Sensitivity Analyses According to Disease-Modifying Therapy

Patients receiving high-efficacy DMT (*n* = 24) had a longer disease duration (*p* = 0.014), a higher baseline EDSS (*p* = 0.015), and more frequent gadolinium-enhancing (*p* = 0.005) and spinal cord lesions (*p* = 0.006) than those receiving moderate-efficacy DMT (*n* = 54), consistent with treatment escalation in patients with more active disease (Supplementary Table S3). During the inter-assessment interval, high-efficacy DMT was associated with fewer patients experiencing relapses (*p* = 0.049), a lower ARR (*p* = 0.050), and a lower frequency of latency prolongation (*p* = 0.012), with a median left-eye latency change of −5 ms versus +1 ms (*p* = 0.004); the frequency of amplitude reduction (*p* = 0.23) and the change in EDSS (*p* = 0.34) did not differ between tiers. The association between latency prolongation and ARR was present within both strata (moderate-efficacy: median ARR 0.66 vs. 0.0, *p* = 0.022; high-efficacy: 1.0 vs. 0.0, *p* = 0.034), and ARR remained associated with latency prolongation after adding DMT tier to the multivariable logistic model (OR 2.73, 95% CI 1.29–5.79, *p* = 0.009; high-efficacy DMT OR 0.44, 95% CI 0.13–1.57, *p* = 0.21) and to the eye-level GEE model (OR 2.39, 95% CI 1.40–4.05, *p* = 0.001). The AUC of longitudinal latency change for concurrent relapse activity was 0.71 in the moderate-efficacy and 0.62 in the high-efficacy stratum. In contrast, the higher EDSS at the second VEP among patients with amplitude reduction was observed in the moderate-efficacy stratum (*p* = 0.007) but not in the smaller high-efficacy stratum (*p* = 0.40). In an exploratory logistic model, high-efficacy DMT was associated with lower odds of amplitude reduction (OR 0.27, 95% CI 0.08–0.87, *p* = 0.028), independent of age, disease duration, baseline EDSS, and ARR. The five untreated patients were too few for separate analysis.

### 3.6. Baseline VEP Abnormalities

At baseline, prolonged P100 latency (>118 ms) was present in 31.3% of patients and was associated with a higher frequency of spinal cord lesions (*p* = 0.032), but not with ARR or EDSS measures at baseline or during follow-up. Reduced baseline amplitude was associated with higher ARR during follow-up (*p* = 0.044), a higher frequency of visual onset symptoms (*p* = 0.029), older age (*p* = 0.045), and fewer gadolinium-enhancing lesions (*p* = 0.015). No significant differences were observed between groups in EDSS values at baseline or at the last assessment (Supplementary Table S1).

## 4. Discussion

In this multicenter retrospective cohort study, we longitudinally evaluated conventional VEP metrics in 83 patients with RRMS. Our findings suggest that longitudinal latency and amplitude changes carry different clinical associations in RRMS. P100 latency prolongation was associated with higher relapse activity during the same observation window, with ARR remaining the sole variable independently associated with latency prolongation in multivariable analysis, and this association was robust to stratification and adjustment for DMT efficacy tier. In contrast, amplitude reduction showed a weaker, group-level association with disability: EDSS at follow-up was 0.5 points higher despite similar baseline scores, but the corresponding correlation analysis yielded only a nominally significant association that did not survive Bonferroni correction for eight simultaneous comparisons, and the group difference was attenuated after adjustment for baseline EDSS. EDSS worsening was not associated with latency changes. Post hoc power analysis further indicated that the study was underpowered for the amplitude–EDSS correlation, so the association requires confirmation in a larger cohort. When a formal definition of PIRA was applied, only three events were identified; although all occurred in patients with amplitude reduction, the number of events is far too small to support any inference, and our data therefore neither establish nor exclude a link between amplitude decline and PIRA. Baseline analyses provided additional context. Prolonged latency was associated with greater spinal cord involvement, whereas reduced amplitude was associated with older age and higher relapse rates; however, neither baseline abnormality was associated with EDSS measures at baseline or during follow-up. In addition, patients with latency prolongation exhibited lower lesion burden and lower CSF IgG index at baseline.

The two principal VEP metrics, P100 latency and amplitude, are generally considered to reflect different pathophysiological aspects of MS. A prolonged P100 latency indicates slowed axonal conduction caused by demyelination along visual pathways [8,11], whereas a reduced P100 amplitude signifies axonal loss or persistent conduction block, reflecting irreversible damage [9,23]. For example, in acute optic neuritis, both latency delay and amplitude attenuation are commonly observed; latency may partially recover with remyelination, but amplitude loss often persists in cases of axonal degeneration [11,23]. These complementary parameters provide a unique window for subclinical demyelination and neurodegeneration. Notably, patients in the progressive phase of MS often exhibit more pronounced abnormalities in both measures, which is consistent with cumulative axonal loss [7,10]. Our findings are compatible with this dual-pathway interpretation, although we emphasize that the present study did not measure inflammation or neurodegeneration directly with independent biomarkers (e.g., gadolinium enhancement during follow-up, serum neurofilament light chain, or OCT), and the mechanistic attribution therefore remains an interpretation of the observed associations rather than a demonstrated conclusion. Latency prolongation was associated with concurrent relapse activity, but not with disability worsening, which is consistent with its known sensitivity to demyelination [8,11]. This association was robust to the non-independence of the two eyes in a GEE model and to adjustment for DMT tier, and yielded moderate discriminative accuracy for concurrent on-study relapse activity, suggesting that serial latency monitoring could serve as a practical correlate of inflammatory activity, pending prospective validation. The weaker, group-level association between amplitude reduction and disability would be in keeping with the concept that axonal loss underlies irreversible functional decline in MS [13,23], but the small effect size, the lack of significance after correction for multiple comparisons, and the attenuation after adjustment for baseline EDSS mean that our data provide only preliminary support for this interpretation. The observation that latency prolongation emerged in patients with fewer spinal cord lesions and a lower IgG index at baseline may suggest that focal optic pathway demyelination may evolve somewhat independently of global disease burden [4,24], although this finding also requires replication. Whether amplitude decline captures ‘silent progression’ or PIRA [21,25] could not be adequately tested here: with a formal PIRA definition, only three events were identified over a median of 2 years, and larger cohorts with longer follow-up will be needed to address this question.

These results are in line with those of previous reports that emphasized the prognostic value of VEP abnormalities in predicting future clinical outcomes. Miletic-Drakulic et al. [14] demonstrated that preserved VEP responses were predictive of achieving NEDA-3 over 10 years, whereas significant unilateral delays were linked to less favorable disease trajectories. Similarly, Vecchio et al. [3] reported that prolonged latency in non-neuritic eyes at diagnosis predicted subsequent disability and lesion accrual. Although baseline VEP abnormalities were not associated with disability worsening in our study, longitudinal changes, particularly amplitude decline, showed a nominal association with concurrent worsening of the EDSS score. These observations are consistent with, but do not prove, an added value of serial monitoring over single-point assessment; the exploratory nature of the correlation findings underscores the need for prospective validation.

Importantly, several classical studies, such as those by Fuhr et al. [15] and Kallmann et al. [16], have shown that early evoked potential abnormalities across multiple modalities can predict long-term outcomes. However, their designs typically rely solely on baseline testing. Our findings raise the possibility that monitoring dynamic changes in VEP parameters over time may offer a more nuanced assessment of the disease course, although the present design cannot establish predictive value. This approach is also supported by more recent work highlighting that VEP metrics correlate with MRI parameters such as lesion volume and brain atrophy, and may outperform standard clinical measures in detecting diffuse or subclinical pathology [6,13]. The reaffirmed diagnostic role of VEP within the 2024 revised McDonald criteria further underscores the value of standardized, serial optic pathway assessment in contemporary MS care [12].

Treatment is an important potential confounder in any observational study of inflammatory activity. In our cohort, patients receiving high-efficacy DMT had more active disease at baseline, in keeping with treatment escalation, yet experienced fewer relapses and less frequent latency prolongation during follow-up, and their left eye latency tended to shorten rather than lengthen. These observations are consistent with reports that effective immunotherapy is accompanied by stabilization or partial recovery of VEP latency [20]. Importantly, the latency-relapse association was present within both DMT strata and persisted after adjustment for DMT tier, arguing against confounding by treatment intensity as the sole explanation for this finding. The amplitude–EDSS association, by contrast, was confined to the moderate-efficacy stratum, and high-efficacy DMT was associated with lower odds of amplitude reduction in an exploratory model. Given the small size of the high-efficacy subgroup, these stratified results should be regarded as hypothesis-generating; nevertheless, they suggest that treatment intensity may influence amplitude evolution and should be accounted for in future prospective studies.

This study has several limitations that should be considered when interpreting its findings. First, the retrospective design and modest sample size constrain generalizability. Post hoc power analysis indicated that the study was adequately powered for the primary logistic regression analysis (power > 99% for OR = 2.91, α = 0.05); however, the correlation analyses were underpowered (power ≈ 56% for ρ = −0.231 at α = 0.05, ≈26% after Bonferroni). Accordingly, the amplitude-related correlation findings should be regarded as hypothesis-generating; prospective studies enrolling at least 145–236 participants will be required to confirm these associations with adequate statistical confidence. Second, the follow-up period (median 2 years) was relatively short for capturing long-term disability trajectories, and the observed between-group EDSS differences (0.5 points) fall below the commonly accepted minimal clinically important difference of approximately 1.0 point, warranting cautious clinical interpretation. Relatedly, because relapses, EDSS change, and VEP change were all ascertained over the same interval, our results describe concurrent associations and cannot establish that VEP changes predict subsequent relapses or disability. Formally defined PIRA events were too few to test the relationship between amplitude decline and relapse-independent progression, and the retrospective design did not allow confirmation of disability worsening at a fixed 3- or 6-month interval. Furthermore, inflammation and neurodegeneration were not measured directly with independent biomarkers, so the pathophysiological interpretation of latency and amplitude changes remains inferential. Third, the amplitude reduction threshold used in this study (a decrease of >0.3 µV from baseline or a value below 5 µV) was operationally defined rather than derived from a validated normative dataset, and its clinical significance relative to established ISCEV criteria requires further evaluation. Fourth, optical coherence tomography (OCT) data were unavailable, precluding structure–function correlation analyses that could have strengthened the interpretation of amplitude changes as a marker of axonal loss [26,27]. In addition, although sensitivity analyses stratified by and adjusted for DMT efficacy tier yielded consistent results for the latency-relapse association, treatment allocation was not randomized, the high-efficacy subgroup was small, and residual confounding by indication (patients with more active disease receiving more potent therapy), by individual agents within each tier, and by treatment duration cannot be excluded; these analyses were not pre-specified and should be considered exploratory. Fifth, although patients with recent optic neuritis were excluded, subclinical episodes occurring between assessments could not be completely ruled out. Sixth, abnormality thresholds were derived from published reference values rather than locally validated norms, which may introduce measurement variability across sites; likewise, although MRI was acquired with a uniform field strength and protocol, images were rated locally rather than by a central reader. Additionally, the use of a relatively large checkerboard check size may have reduced protocol sensitivity compared with the smaller check sizes recommended in current ISCEV guidelines, potentially underestimating the prevalence of subclinical demyelination. Finally, the independent analysis of each eye may introduce intra-subject correlation effects that we addressed in a sensitivity analysis using a generalized estimating equation model, which confirmed the latency-relapse association; nonetheless, larger cohorts are needed to extend such eye-level models to disability outcomes. Prospective multimodal studies with larger cohorts, extended follow-up, locally established reference values, and pre-specified correction for multiple comparisons are warranted to confirm and extend these findings.

## 5. Conclusions

In this exploratory multicenter retrospective cohort, longitudinal P100 latency prolongation was independently associated with concurrent relapse activity, and this association was robust to adjustment for disease-modifying therapy, whereas amplitude decline showed only a weak group-level association with disability that did not survive correction for multiple comparisons and was attenuated after adjustment for baseline EDSS. These findings are compatible with, but do not demonstrate, distinct inflammatory and neurodegenerative substrates for the two VEP parameters, and the small number of formally defined PIRA events precluded any conclusion regarding relapse-independent progression. Serial VEP assessment may merit further evaluation as a complementary tool within multimodal MS monitoring, but prospective studies with larger cohorts, longer follow-up, and independent biomarkers of inflammation and neurodegeneration are required before longitudinal VEP changes can inform individualized management decisions.

## Figures and Tables

**Table 1:** Baseline demographic, clinical, and radiological characteristics of the study population (*n* = 83).

Variable	Value
Demographics	
Age, years	36 (18–60)
Female sex, *n* (%)	66 (79.5)
Disease duration, years	6.0 (1.3–25.2)
Clinical characteristics	
EDSS score at baseline	1.5 (0–5.5)
Annualized relapse rate (ARR)	0.3 (0–3.0)
Interval between VEPs, years	2.0 (1–6)
Baseline VEP parameters	
P100 latency, ms (right/left)	116 (95–162)/114 (96–168)
Amplitude, µV (right/left)	9.0 (0.2–20.5)/9.5 (1.8–20.5)
Baseline MRI findings, *n* (%)	
Brainstem lesion	27 (32.5)
Spinal cord lesion	48 (57.8)
Upper cervical cord lesion (C1–C4)	30 (36.1)
T2 lesion count ≥ 9	46 (55.4)
Gadolinium-enhancing lesion	32 (38.6)
CSF findings	
Oligoclonal bands positive *, *n* (%)	56/72 (77.8)
IgG index	0.8 (0.4–2.2)
Disease-modifying therapy, *n* (%)	
Platform/moderate-efficacy	54 (65.1)
High-efficacy	24 (28.9)
Untreated	5 (6.0)

Abbreviations: VEP, visual evoked potentials; EDSS, Expanded Disability Status Scale; MRI, magnetic resonance imaging; CSF, cerebrospinal fluid. * Available in 72 patients.

**Table 2:** Clinical and paraclinical characteristics according to longitudinal P100 latency changes during follow-up.

Variable	No Latency Prolongation (*n* = 47)	Latency Prolongation (*n* = 36)	*p* Value
Demographics			
Age, years	36 (23–60)	36 (18–58)	0.993
Female sex, *n* (%)	36 (76.6)	30 (83.3)	0.632
Clinical features			
Disease duration, years	7.0 (1.3–25.2)	5.0 (1.6–14.2)	0.062
EDSS at baseline	1.5 (0–5.5)	1.5 (1.0–3.5)	0.521
EDSS at follow-up	1.5 (0–6.5)	1.5 (1.0–3.5)	0.422
EDSS at last visit	1.5 (0–6.5)	1.5 (1.0–3.5)	0.648
ARR	0.0 (0–2.0)	0.9 (0–3.0)	<0.001
Baseline MRI findings, *n* (%)			
Brainstem lesion	20 (42.6)	7 (19.4)	0.047
Spinal cord lesion	34 (72.3)	14 (38.9)	0.005
Upper cervical cord lesion (C1–C4)	22 (46.8)	8 (22.2)	0.038
Gadolinium-enhancing lesion	25 (53.2)	7 (19.4)	0.004
CSF findings			
OCB positivity *, *n* (%)	32/39 (82.1)	24/33 (72.7)	0.507
IgG index	0.9 (0.5–2.2)	0.7 (0.4–1.4)	0.035

Abbreviations: EDSS, Expanded Disability Status Scale; MRI, magnetic resonance imaging; ARR, annualized relapse rate; CSF, cerebrospinal fluid; OCB, oligoclonal bands. * Available in 72 patients. Latency prolongation was defined as an increase of ≥2 ms from baseline in at least one eye.

**Table 3:** Clinical and paraclinical characteristics according to longitudinal P100 amplitude reduction during follow-up.

Variable	No Amplitude Reduction (*n* = 36)	Amplitude Reduction (*n* = 47)	*p* Value
Demographics			
Age, years	37 (23–58)	36 (18–60)	0.639
Female sex, *n* (%)	31 (86.1)	35 (74.5)	0.304
Clinical features			
Disease duration, years	5.3 (1.5–25.2)	6.0 (1.3–20.0)	0.266
ARR	0.0 (0–2.0)	0.3 (0–3.0)	0.849
Baseline VEP parameters			
P100 latency, ms (right eye)	119.5 (96.6–162)	112 (95–151)	0.028
Disability outcomes			
EDSS at baseline	1.0 (0–4.5)	1.5 (1–5.5)	0.118
EDSS at follow-up VEP	1.0 (0–5.0)	1.5 (1–6.5)	0.017
EDSS at last follow-up	1.0 (0–5.0)	1.5 (0–6.5)	0.028
Baseline MRI findings, *n* (%)			
Brainstem lesion	12 (33.3)	15 (31.9)	1.000
Spinal cord lesion	20 (55.6)	28 (59.6)	0.886
Gadolinium-enhancing lesion	15 (41.7)	17 (36.2)	0.778
CSF findings			
OCB positivity *, *n* (%)	25/31 (80.6)	31/41 (75.6)	0.824
IgG index	0.8 (0.5–2.2)	0.8 (0.4–2.1)	0.722

Abbreviations: EDSS, Expanded Disability Status Scale; MRI, magnetic resonance imaging; CSF, cerebrospinal fluid; OCB, oligoclonal bands; ARR, annualized relapse rate; VEP, visual evoked potentials. * Available in patients with CSF data. Amplitude reduction was defined as a decrease of >0.3 µV from baseline or as a value below 5 µV in at least one eye.

**Table 4:** Correlation between longitudinal changes in VEP parameters and disability worsening (EDSS).

VEP Parameter	EDSS Change (2nd VEP–1st VEP)	EDSS Change (Final–2nd VEP)
P100 latency		
Right eye	ρ = −0.156, *p* = 0.160	ρ = 0.009, *p* = 0.936
Left eye	ρ = −0.158, *p* = 0.154	ρ = −0.140, *p* = 0.208
P100 amplitude		
Right eye	ρ = −0.140, *p* = 0.208	ρ = 0.019, *p* = 0.864
Left eye	ρ = −0.231, *p* = 0.036	ρ = 0.019, *p* = 0.866

Abbreviations: VEP, visual evoked potentials; EDSS, Expanded Disability Status Scale.

## Data Availability

The data presented in this study are available on request from the corresponding author. The anonymized minimal dataset supporting the reported findings can also be provided to the editorial office as Supporting Information for internal evaluation upon request.

## Supplementary Materials

The following supporting information can be downloaded from https://www.mdpi.com/article/10.3390/medicina62091750/s1: Supplementary Table S1 (Baseline VEP abnormalities and their clinical associations), Supplementary Table S2 (Distribution of disease-modifying therapies used between VEP assessments (*n* = 83)), and Supplementary Table S3 (Clinical characteristics, relapse activity, and longitudinal VEP outcomes according to disease-modifying therapy efficacy tier (*n* = 78; five untreated patients excluded)).

## References

[1] Hojjati S.M., Zarghami A., Hojjati S.A., Baes M. (2015). Optic neuritis, the most common initial presenting manifestation of multiple sclerosis in northern Iran. Casp. J. Intern. Med..

[2] Miller D., Barkhof F., Montalban X., Thompson A., Filippi M. (2005). Clinically isolated syndromes suggestive of multiple sclerosis, part I: Natural history, pathogenesis, diagnosis, and prognosis. Lancet Neurol..

[3] Vecchio D., Barbero P., Galli G., Virgilio E., Naldi P., Comi C., Cantello R. (2023). Prognostic Role of Visual Evoked Potentials in Non-Neuritic Eyes at Multiple Sclerosis Diagnosis. J. Clin. Med..

[4] Martinez-Lapiscina E.H., Arnow S., A Wilson J., Saidha S., Preiningerova J.L., Oberwahrenbrock T., Brandt A.U., E Pablo L., Guerrieri S., Gonzalez I. (2016). Retinal thickness measured with optical coherence tomography and risk of disability worsening in multiple sclerosis: A cohort study. Lancet Neurol..

[5] Klistorner A., Graham E.C., Yiannikas C., Barnett M., Parratt J., Garrick R., Wang C., You Y., Graham S.L. (2017). Progression of retinal ganglion cell loss in multiple sclerosis is associated with new lesions in the optic radiations. Eur. J. Neurol..

[6] Oertel F.C., Krämer J., Motamedi S., Keihani A., Zimmermann H.G., Dimitriou N.G., Condor-Montes S., Bereuter C., Cordano C., Abdelhak A. (2023). Visually Evoked Potential as Prognostic Biomarker for Neuroaxonal Damage in Multiple Sclerosis From a Multicenter Longitudinal Cohort. Neurol. Neuroimmunol. Neuroinflamm..

[7] Guerrieri S., Comi G., Leocani L. (2021). Optical coherence tomography and visual evoked potentials as prognostic and monitoring tools in progressive multiple sclerosis. Front. Neurosci..

[8] Odom J.V., Bach M., Brigell M., Holder G.E., McCulloch D.L., Mizota A., Tormene A.P., International Society for Clinical Electrophysiology of Vision (2016). ISCEV standard for clinical visual evoked potentials: (2016 update). Doc. Ophthalmol..

[9] Leocani L., Guerrieri S., Comi G. (2018). Visual evoked potentials as a biomarker in multiple sclerosis and associated optic neuritis. J. Neuroophthalmol..

[10] Leocani L., Rovaris M., Boneschi F.M., Medaglini S., Rossi P., Martinelli V., Amadio S., Comi G. (2006). Multimodal evoked potentials to assess the evolution of multiple sclerosis: A longitudinal study. J. Neurol. Neurosurg. Psychiatry.

[11] Klistorner A., Arvind H., Nguyen T., Garrick R., Paine M., Graham S., O’DAy J., Grigg J., Billson F., Yiannikas C. (2008). Axonal loss and myelin in early ON loss in postacute optic neuritis. Ann. Neurol..

[12] Saidha S., Green A.J., Leocani L., Vidal-Jordana A., Kenney R.C., Bsteh G., Outteryck O., Thompson A., Montalban X., Coetzee T. (2025). The use of optical coherence tomography and visual evoked potentials in the 2024 McDonald diagnostic criteria for multiple sclerosis. Lancet Neurol..

[13] Covey T.J., Golan D., Zarif M., Bumstead B., Buhse M., Kaczmarek O., Sergott R., Wilken J., Sima D.M., Van Hecke W. (2022). Individual differences in visual evoked potential latency are associated with variance in brain tissue volume in people with multiple sclerosis: An analysis of brain function-structure correlates. Mult. Scler. Relat. Disord..

[14] Miletic-Drakulic S., Miloradovic I., Jankovic V., Azanjac-Arsic A., Lazarevic S. (2022). VEP Score of a Left Eye Had Predictive Values for Achieving NEDA-3 over Ten Years in Patients with Multiple Sclerosis. Sensors.

[15] Fuhr P., Borggrefe-Chappuis A., Schindler C., Kappos L. (2001). Visual and motor evoked potentials in the course of multiple sclerosis. Brain.

[16] Kallmann B.A., Fackelmann S., Toyka K.V., Rieckmann P., Reiners K. (2006). Early abnormalities of evoked potentials and future disability in patients with multiple sclerosis. Mult. Scler..

[17] Thompson A.J., Banwell B.L., Barkhof F., Carroll W.M., Coetzee T., Comi G., Correale J., Fazekas F., Filippi M., Freedman M.S. (2018). Diagnosis of multiple sclerosis: 2017 revisions of the McDonald criteria. Lancet Neurol..

[18] A Samjoo I., Worthington E., Drudge C., Zhao M., Cameron C., A Häring D., Stoneman D., Klotz L., Adlard N. (2021). Efficacy classification of modern therapies in multiple sclerosis. J. Comp. Eff. Res..

[19] Montalban X., Gold R., Thompson A.J., Otero-Romero S., Amato M.P., Chandraratna D., Clanet M., Comi G., Derfuss T., Fazekas F. (2018). ECTRIMS/EAN guideline on the pharmacological treatment of people with multiple sclerosis. Mult. Scler..

[20] Saridas F., Hojjati F., Alizada S., Lazrak S., Koc E.R., Turan O.F. (2024). Effects of disease-modifying therapies on remyelination in multiple sclerosis; evaluation via visual evoked potential test. Mult. Scler. Relat. Disord..

[21] Kappos L., Wolinsky J.S., Giovannoni G., Arnold D.L., Wang Q., Bernasconi C., Model F., Koendgen H., Manfrini M., Belachew S. (2020). Contribution of relapse-independent progression vs relapse-associated worsening to overall confirmed disability accumulation in typical relapsing multiple sclerosis in a pooled analysis of 2 randomized clinical trials. JAMA Neurol..

[22] Lublin F.D., A Häring D., Ganjgahi H., Ocampo A., Hatami F., Čuklina J., Aarden P., Dahlke F., Arnold D.L., Wiendl H. (2022). How patients with multiple sclerosis acquire disability. Brain.

[23] You Y., Klistorner A., Thie J., Gupta V.K., Graham S.L. (2012). Axonal loss in a rat model of optic neuritis is closely correlated with visual evoked potential amplitudes using electroencephalogram-based scaling. Investig. Ophthalmol. Vis. Sci..

[24] Alshowaeir D., Yiannikas C., Klistorner A. (2015). Multifocal visual evoked potential (mfVEP) and pattern-reversal visual evoked potential changes in patients with visual pathway disorders: A case series. Neuroophthalmology.

[25] Giovannoni G., Butzkueven H., Dhib-Jalbut S., Hobart J., Kobelt G., Pepper G., Sormani M.P., Thalheim C., Traboulsee A., Vollmer T. (2016). Brain health: Time matters in multiple sclerosis. Mult. Scler. Relat. Disord..

[26] Tavazzi E., Jakimovski D., Kuhle J., Hagemeier J., Ozel O., Ramanathan M., Barro C., Bergsland N., Tomic D., Kropshofer H. (2020). Serum neurofilament light chain and optical coherence tomography measures in MS: A longitudinal study. Neurol. Neuroimmunol. Neuroinflamm..

[27] Petzold A., Balcer L.J., Calabresi P.A., Costello F., Frohman T.C., Frohman E.M., Martinez-Lapiscina E.H., Green A.J., Kardon R., Outteryck O. (2017). Retinal layer segmentation in multiple sclerosis: A systematic review and meta-analysis. Lancet Neurol..

