# Supplementary material for: Longitudinal Visual Evoked Potential Changes Show Distinct Associations with Relapse Activity and Disability in Relapsing–Remitting Multiple Sclerosis: A Multicenter Retrospective Cohort Study
Source: Medicina (Kaunas). 2026 Sep 11;62(9):1750. doi: 10.3390/medicina62091750 (PMC13609181; doi:10.3390/medicina62091750)
Supplement: Supplementary file 1 [file medicina-62-01750-s001.zip › medicina-4490130-supplementary.pdf]

Supplementary Materials

Supplementary Table S1. Baseline VEP abnormalities and their clinical associations.

Variable	Normal baseline	Abnormal baseline	p value
P100 latency ≤118 ms vs >118 ms	(n = 57)	(n = 26)	
Spinal cord lesion, n (%)	28 (49.1)	20 (76.9)	0.032
ARR during follow-up	0.3 (0–2.0)	0.2 (0–3.0)	0.566
EDSS at baseline	1.5 (0–5.5)	1.5 (0–5.5)	0.231
EDSS at last follow-up	1.5 (0–6.5)	1.5 (0–6.5)	0.303
Amplitude normal vs reduced	(n = 65)	(n = 18)	
Age, years	36 (18–60)	40.5 (23–58)	0.045
Visual onset, n (%)	17 (26.2)	10 (55.6)	0.029
ARR during follow-up	0.0 (0–3.0)	1.0 (0–2.0)	0.044
Gd+ lesion, n (%)	30 (46.2)	2 (11.1)	0.015
EDSS at baseline	1.5 (0–6.0)	1.0 (1–3.0)	0.254
EDSS at last follow-up	1.5 (0–6.5)	1.0 (1.0–6.5)	0.213

Abbreviations: EDSS, Expanded Disability Status Scale; ARR, annualized relapse rate; Gd+, gadolinium-enhancing. Values are median (range) unless stated otherwise.

Supplementary Table S2. Distribution of disease-modifying therapies used between VEP assessments (n = 83).

Disease-modifying therapy	n (%)
Dimethyl fumarate	19 (22.9)
Teriflunomide	16 (19.3)
Fingolimod	11 (13.3)
Glatiramer acetate	10 (12.0)
Interferon beta	9 (10.8)
Ocrelizumab	7 (8.4)
Natalizumab	5 (6.0)
Untreated	5 (6.0)
Cladribine	1 (1.2)

Supplementary Table S3. Clinical characteristics, relapse activity, and longitudinal VEP outcomes according to disease-modifying therapy efficacy tier (n = 78; five untreated patients excluded).

Variable	Moderate-efficacy DMT (n = 54)	High-efficacy DMT (n = 24)	p value
Baseline characteristics			
Age, years	36.0 (18–60)	36.5 (23–58)	0.685
Female sex, n (%)	41 (75.9)	20 (83.3)	0.562
Disease duration, years	5.2 (1.3–25.2)	8.0 (1.6–20.0)	0.014
EDSS at first VEP	1.5 (0–4.5)	2.0 (1.0–5.5)	0.015
Spinal cord lesion, n (%)	27 (50.0)	20 (83.3)	0.006

Variable	Moderate-efficacy DMT (n = 54)	High-efficacy DMT (n = 24)	p value
Gadolinium-enhancing lesion, n (%)	14 (25.9)	15 (62.5)	0.005
Interval between VEPs, years	2.1 (1.0–4.4)	1.3 (1.0–6.0)	0.285
Relapse activity during the interval			
ARR	0.4 (0–3.0)	0.0 (0–2.0)	0.050
≥1 relapse, n (%)	32 (59.3)	8 (33.3)	0.049
Longitudinal VEP outcomes			
Latency prolongation, n (%)	29 (53.7)	5 (20.8)	0.012
Amplitude reduction, n (%)	33 (61.1)	11 (45.8)	0.227
P100 latency change, right eye, ms	0.0 (−22 to 36)	−3.0 (−25 to 14)	0.059
P100 latency change, left eye, ms	1.0 (−18 to 34)	−5.0 (−48 to 8)	0.004
Amplitude change, right eye, μV	0.5 (−7.4 to 14.0)	0.3 (−9.5 to 11.0)	0.729
Amplitude change, left eye, μV	−0.3 (−7.4 to 10.5)	0.1 (−11.5 to 7.5)	0.488
Disability outcomes			
EDSS at second VEP	1.5 (0–5.5)	2.0 (1.0–6.0)	0.048
EDSS change (2nd − 1st VEP)	0.0 (−2.0 to 4.0)	0.0 (−1.5 to 1.5)	0.339
Confirmed disability worsening, n (%)	5 (9.3)	2 (8.3)	1.000
PIRA, n (%)	1 (1.9)	1 (4.2)	0.523
Associations within each DMT stratum			
ARR: latency prolongation vs none (median)	0.66 vs 0.0; p = 0.022	1.0 vs 0.0; p = 0.034	
EDSS at 2nd VEP: amplitude reduction vs none (median)	1.5 vs 1.0; p = 0.007	2.0 vs 2.0; p = 0.403	
AUC of latency change for on-study relapse activity	0.71	0.62	

Moderate-efficacy DMT: interferon-β, glatiramer acetate, teriflunomide, dimethyl fumarate. High-efficacy DMT: fingolimod, natalizumab, cladribine, ocrelizumab. Values are median (range) unless stated otherwise. Continuous variables were compared with the Mann–Whitney U test and categorical variables with Fisher's exact test. Latency prolongation: increase of ≥2 ms from baseline in at least one eye; amplitude reduction: decrease of >0.3 μV from baseline in at least one eye. Confirmed disability worsening: EDSS increase of ≥1.5 (baseline 0), ≥1.0 (baseline 1.0–5.5) or ≥0.5 (baseline >5.5) at the second VEP, sustained at the last visit; PIRA: confirmed worsening without any relapse during the interval. In multivariable logistic regression including age, disease duration, baseline EDSS, ARR and DMT tier, ARR remained associated with latency prolongation (OR 2.73, 95% CI 1.29–5.79, p = 0.009; high-efficacy DMT OR 0.44, 95% CI 0.13–1.57, p = 0.207); in the eye-level GEE model, ARR OR 2.39 (95% CI 1.40–4.05, p = 0.001). Abbreviations: ARR, annualized relapse rate; AUC, area under the receiver-operating-characteristic curve; DMT, disease-modifying therapy; EDSS, Expanded Disability Status Scale; GEE, generalized estimating equation; PIRA, progression independent of relapse activity; VEP, visual evoked potential.
